# Internet addiction in times of COVID-19: Review of the situation

**DOI:** 10.1192/j.eurpsy.2021.1757

**Published:** 2021-08-13

**Authors:** M. Regaya, W. Abdelghaffar, B. Amamou, R. Rafrafi, L. Gaha

**Affiliations:** 1 Department Of Psychiatry, University of Monastir, Faculty of Medicine of Monastir, LR05ES10, Fattouma Bourguiba Hospital, Monastir, Tunisia; 2 Psychiatry Department, General Hospital Mongi Slim, Marsa, Tunisia

**Keywords:** Internet, Addiction, COVID-19

## Abstract

**Introduction:**

Covid-19 pandemic represents a sanitary crisis with social, economic and political negative impacts. Studies had well established the link between stress and the display or maintenance of addictive behaviour. Measures like social distancing and confinement imposed by governments during this pandemic, could explain an outbreak of internet addiction cases.

**Objectives:**

Assess the prevalence of internet addiction in times of the covid-19 pandemic and to study its relationship with personal and family factors but also with the psychiatric comorbidities.

**Methods:**

We conducted a cross sectional analytical study during the period of the covid-19 pandemic, using an internet survey exploring socio-demographic and clinical data: through Hamilton depression and anxiety rating scale and the Rosenberg self esteem scale. The assessment of internet addiction was carried out using Young’s Internet Addiction test.

**Results:**

Our study included 150 users, the average age was 36 years, mainly made up of women, married, having children, with a good socioeconomic level and of university education. The prevalence of depressive and anxious symptoms were respectively 7.3% and 18.7%. The prevalence of internet addiction in our study was 9.4% including 0.7% having severe repercussions. Being married (p<10-3) and having children (p=0.006) were considered as protective factors against internet addiction. People having an internet addiction were at bigger risk of having low self esteem (p=0.023), depressive (p=0.04) and anxious symptoms (p<10-3).

**Conclusions:**

Internet addiction is a new concept, though it’s impact on the well being is well established and it’s likely to worsen in times of pandemic. Therefore, it’s necessary to take preventive measures to deal with it.

**Disclosure:**

No significant relationships.

